# Supplementary material for: Reinforcement learning in cold atom experiments
Source: Nat Commun. 2024 Oct 2;15:8532. doi: 10.1038/s41467-024-52775-8 (PMC11447118; doi:10.1038/s41467-024-52775-8)
Supplement: Supplementary file 2 — Description of Additional Supplementary Files [file 41467_2024_52775_MOESM2_ESM.pdf]

Description of Additional Supplementary Files

File Name: Supplementary Movie 1

Description: Fluorescence images of the MOT over the course of four different episodes, corresponding to insets in Fig. 1d. The data correspond to episodes of in-training evaluation performed with a random offset applied to the control parameter. The shown fluorescence images are used as input to the agent, trained to maximize N/T in the MOT. Note, that the fluorescence intensity does not directly correspond to the number of atoms, as it also depends on the applied laser detuning.
